# The applicability of fluorescent optotracers for in vitro and in vivo* Staphylococcus aureus* detection and quantification

**DOI:** 10.1038/s41598-025-17029-7

**Published:** 2025-10-03

**Authors:** Liliana Agresti, Elles C. Boonstra, Paul C. Jutte, Henny C. van der Mei, Jelmer Sjollema

**Affiliations:** 1 University of Groningen, University Medical Center Groningen, Department of Biomaterials & Biomedical Technology, Antonius Deusinglaan 1, 9713 AV Groningen, The Netherlands; 2 University of Groningen, University Medical Center Groningen, Department of Orthopaedics, Hanzeplein 1, 9700 RB Groningen, The Netherlands

**Keywords:** Optotracers, Bacterial detection, Biofilms, Bioluminescent bacteria, Probe specificity, In vivo imaging of infection, Microbiology, Applied microbiology, Applied optics, Diagnostic markers

## Abstract

A sensitive and specific method for assessing microbial contamination is crucial in many sectors of our society like the medical field. Optotracers that trigger fluorescence upon binding to bacterial cell surfaces offer a novel approach. Several studies have highlighted limitations in the specificity of these optotracers with respect to their molecular targets, but, to the best of our knowledge, none did in vivo studies with the same bacterial strain as the in vitro studies. In this study, we compared the activatable optotracer EbbaBiolight 680 for bacterial detection, both in vitro and in vivo with the same *Staphylococcus aureus* bacterial strain*,* while analyzing the sensitivity and specificity of the probe against this strain. In vitro the probe’s fluorescence correlated strongly with the number of bacterial colony-forming units, both in planktonic suspension and biofilms. However, in vivo results from a mouse model demonstrated limited specificity for *S. aureus*, as the probe also binds to repetitive component motifs in the extracellular matrix of the tissue. This resulted in a substantial background signal that obscured bacterial detection. In conclusion, while EbbaBiolight 680 effectively detects *S. aureus* in planktonic suspension and biofilms in vitro, the probe has unfortunately limited specificity in vivo, which can hinder accurate bacterial detection.

## Introduction

Controlling the appearance of microbial contamination is essential in many sectors of our society like industrial quality control for food and drugs, a sector in which bacterial contamination must be avoided^1,2^. Furthermore, controlling or preventing bacterial infections also plays a pivotal role in the biomedical field, e.g. in wound infections as well as in biomaterial-associated infections following biomaterial implantation for medical purposes. This type of infection, associated with bacterial biofilm formation, can lead to the failure of biomaterial implants, often requiring subsequent replacement or surgical removal. *Staphylococcus aureus* is frequently involved in these biomaterial-associated infections^3–6^.

*S. aureus* is one of the most widespread pathogenic bacteria capable of invading various animal hosts including humans and causes a range of diseases from skin infections to biomaterial-associated infections and severe sepsis^7–10^. For studying the course of infection by pathogens, often animal models are used with bacterial strains that are genetically modified, e.g. by the integration of the *Photorhabdus luminescens* luxABCDE operon into the bacterial plasmid, resulting in bioluminescence production^11–13^. The intensity of bioluminescence is a powerful measure of the metabolic activity of bacteria and correlates with the number of colony forming units (CFUs)^14^. Therefore, bioluminescence can be used to monitor the course of infection, evaluate the efficacy of antimicrobial therapies and assess the influence of the microbial environment on the metabolic activity of microorganisms^15,16^. However, detection of bioluminescence needs dedicated and sensitive instrumentation which usually lacks sufficient resolution to visualize individual bacteria.

In the case of infection control in clinical applications, optotracers can be used for bacterial detection. Optotracers activate fluorescence when binding to a target, enabling real-time and non-invasive detection. Optotracers are small anionic fluorescent tracer molecules classified as luminescent conjugated oligothiophenes^17,18^. These organic molecules utilize electrostatic interactions to bind to target molecules such as peptides and carbohydrate-based biopolymers. Upon binding, the tracers adopt a conformation that enhances and switches-on strong fluorescence. The application of optotracers allows for the real-time monitoring of the bacterial proliferation and the production of extracellular polymeric substances (EPS) within biofilms^19^.

Recently a probe, based on this optotracer paradigm, has been introduced as EbbaBiolight 680 (EL) (Ebba Biotech AB, Stockholm, Sweden). This probe is activated upon binding to the bacterial cell wall, specifically to peptidoglycan and lipoteichoic acid, acting in a limited frequency band-width that provides a fingerprint for the identification of the target biopolymer^20,21^. Several studies have used these probe characteristics and in particular the probe activatability to carry out in vitro bacterial detection of *S. aureus*^21,22^. Probe activation by a selective target avoids washing procedures when used in vitro and may simplify interpretation when used in vivo, since often inflammation and infection both allow accumulation of a probe. Previously, we investigated the use of an activatable probe, a fluorophore conjugated to a fluorescent quencher that could be cleaved by a nuclease enzyme upon which the fluorophore is activated. This enzyme is an actively expressed component by *S. aureus* and plays a role in biofilm formation^23^. However, in our animal infection model, we were unable to establish a correlation between the number of colony-forming units (CFUs) and fluorescence^24^. Therefore, we investigated the applicability of the activatable EL probe for fluorescence imaging of bacterial infections both in vitro and in vivo.

To this end, we evaluated the sensitivity and specificity of the probe towards *S. aureus* Xen36 under various conditions. We investigated the availability of the tracer during bacterial growth, the correlation of the fluorescent intensity with CFUs, both for planktonic bacteria as well as for bacteria in a biofilm. Finally, we related the use of the optotracer in an in vivo infection mouse model using macroscopic imaging and two-photon microscopy.

## Results

### The sensitivity of the EL probe for bacteria suspended in buffer

**Fig. 1:**
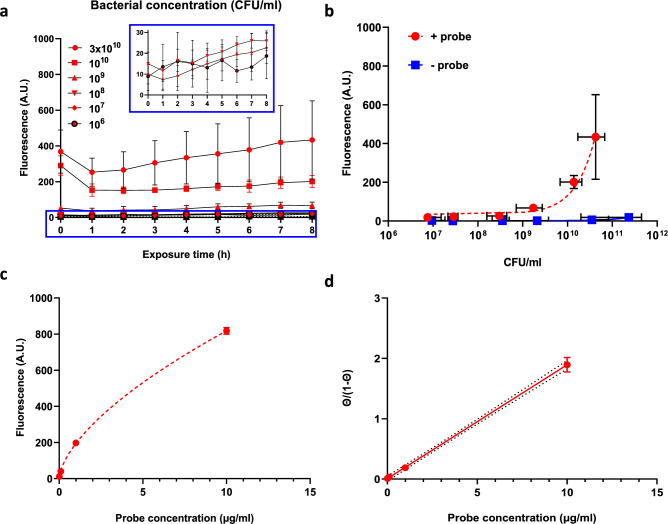
Fluorescence intensity of the EL probe as a function of bacterial and probe concentrations. (**a**) Fluorescence of planktonic bacteria in PBS with 2% TSB with the EL probe (1 µg/ml) incubated as a function of time with various bacterial concentrations. (Insert: Fluorescence intensity of the EL probe for CFU concentrations from 10^6^ to 10^8^ CFU/ml). The controls depicted as a dotted line represent the same bacterial concentrations without the probe. The controls are below the detection limit of the plate reader (n = 3, biological replicates). (**b**) Same as panel a, now as a function of the concentration of CFUs after 8 h. The dotted red line is a regression line (n = 3, biological replicates). (**c**) Fluorescence from a bacterial suspension with a concentration of 10^10^ CFU/ml as a function of the EL probe concentration. The dotted line is a regression line to indicate non-linearity. (**d**) Langmuir adsorption plot based on the data of c), according to Eq. 1 with Γ_max_ = 1250 A.U. The dotted line represents a linear regression line fitted using Eq. 2 (R_squared_ = 0.996).

The fluorescence intensity of the EL probe, measured for different bacterial concentrations in phosphate buffered saline (PBS) with 2% tryptone soya broth (TSB), was immediately acquired at t = 0 and remained constant over an 8 h time period (Fig. 1a). The fluorescence intensities were significantly higher (P < 0.0004) than the controls without the EL probe (Fig. 1a), although the assay is not sensitive enough to significantly distinguish the three lowest concentrations (10^6^, 10^7^ and 10^8^). Additionally, Fig. 1b shows that below 10^8^ CFU/ml fluorescence was rather constant, following the regression line based on Eq. 1 (see Materials and methods section), indicating a low fluorescent background caused by non-adsorbed tracers (see also Supplementary material Fig. S1). The increase in fluorescence for the assay without the probe is caused by the bacteria which exhibit slight autofluorescence.

In order to further explore the relationship between the EL probe concentration and fluorescence intensity, one bacterial concentration (10^10^ CFU/ml) was used with various EL probe concentrations. Figure 1c shows the typical shape of an adsorption isotherm and in Fig. 1d the data were fitted to a linear function as presented in Eq. 2 (see Materials and methods section), with $\frac{\theta }{1-\theta }$ indicating the ratio of the percentage of bound EL probe (θ) and the percentage of unbound EL probe (1-θ), showing that the adsorption of the probe follows a Langmuir-type of adsorption, which involves both desorption from binding sites and competitive binding (or blocking).

### EL probe sensitivity for planktonic and biofilm bacteria during growth

**Fig. 2:**
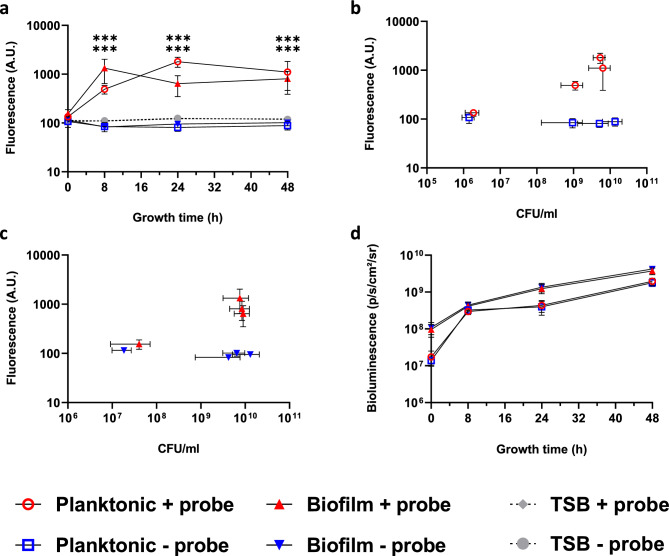
Fluorescence and bioluminescence during bacterial growth in a planktonic and biofilm mode of growth. (**a**) Fluorescence of the EL probe (1 μg/ml) for planktonic and biofilm bacteria as a function of growth time. (**b**) Fluorescence of the EL probe on the bacteria as a function of the CFUs of the planktonic bacterial concentration taken at the same time points as in panel a. (**c**) Same as panel b, now for biofilm bacteria. (**d**) Bioluminescent radiance as a function of growth for planktonic and biofilm bacteria. Statistical differences were calculated using multiple t-tests and the differences between planktonic and biofilm mode of growth with EL probe compared to the control without the probe were considered statistically significant when P < 0.05, *** P < 0.001, (n = 3, biological replicates).

*S. aureus* Xen36 was grown planktonically and in the biofilm mode in the presence of the EL probe (1 µg/ml). An increase of fluorescence was observed during the first 24 h of growth after which a stable plateau was reached up to 48 h for planktonically grown *S. aureus* Xen36. In contrast, the biofilm mode of growth showed an increase only during the first 8 h (Fig. 2a). No difference was observed between the fluorescence of the EL probe bound to bacteria grown planktonically or in a biofilm. A statistically significant difference (P < 0.001), was observed between the samples with the probe and the negative controls without the probe. The initial inoculum for *S. aureus* Xen36 was 10^6^ CFU/ml, but further analyses were also conducted for a starting inoculum of 10^8^ CFU/ml which showed similar results (Supplementary material, Fig. S2).

In order to verify the probe sensitivity and specificity with respect to other bacterial strains we measured the fluorescence from two other Gram-positive strains (*S. aureus* ATCC12600 and *Staphylococcus epidermidi*s ATCC 35984) and the Gram-negative *Escherichia coli* ATCC 25922 (Supplementary material, Fig. S3). The results showed clearly the selectivity of the probe for Gram-positive strains.

To investigate the relationship between CFUs and the fluorescent signal of the EL probe, fluorescence was reported *versus* the number of CFUs from the same bacterial culturing assays, both for planktonic (Fig. 2b) and biofilm mode of growth (Fig. 2c). The fluorescence for the different bacterial concentrations compared to the controls appeared significantly higher (P < 0.001) only when the number of CFU/ml was equal to or larger than 10^9^ bacteria/ml. Although the number of CFUs increased 4 decades during culturing (from 10^6^ to 10^10^ CFUs), the fluorescence increased only from 10^2^ to 10^3^.

The metabolic activity of the planktonically and biofilm grown bacteria was measured using bioluminescence. No considerable difference was observed between the CFU/ml of the planktonic and biofilm bacteria with or without the EL probe (Fig. 2b,c). Bioluminescence increased 2 decades during incubation time, much less than the increase in CFU numbers (4 decades), but higher than the one of EL fluorescence (1 decade) (Fig. 2d). Additionally, bioluminescence did not reach a clear plateau, unlike the fluorescence observed with the EL probe.

### Probe specificity to bacteria

**Fig. 3:**
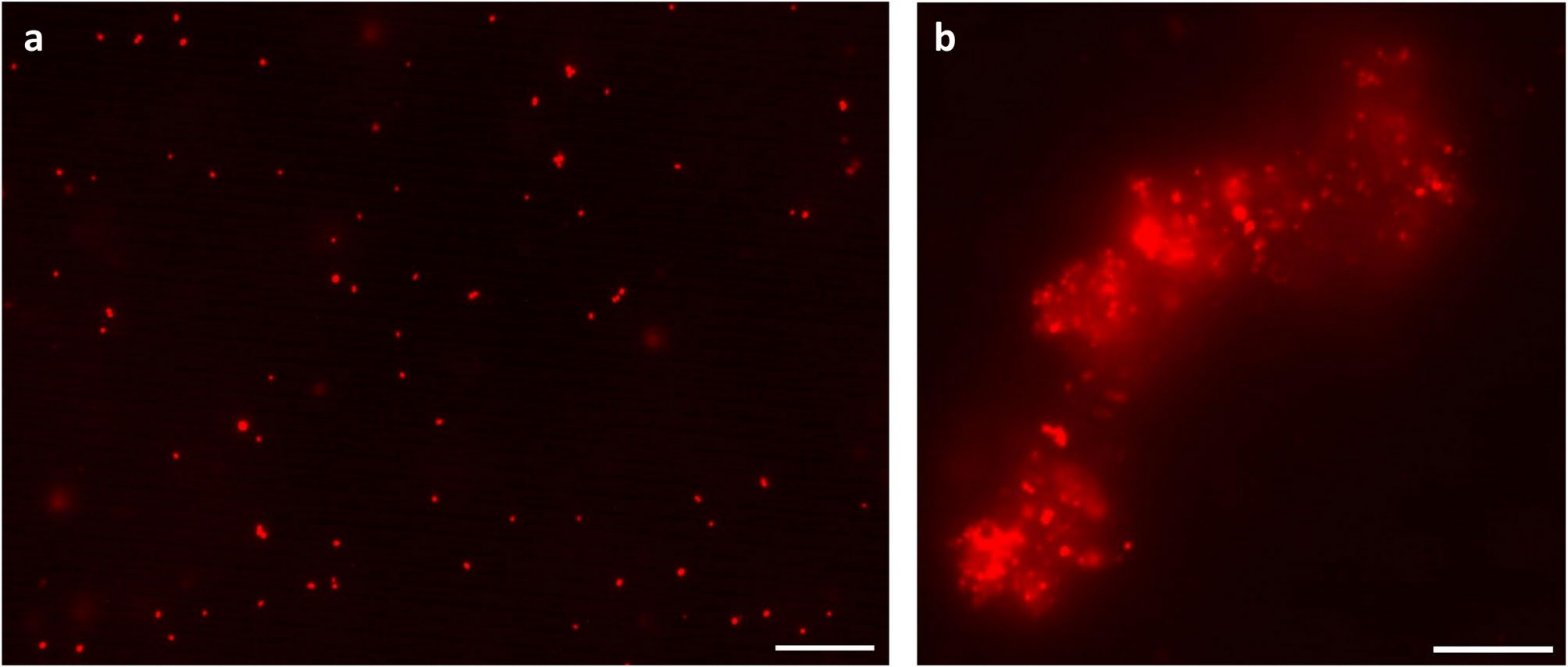
Evaluation of EL probe specificity with fluorescence microscopy. (**a**) Staining of single S. aureus Xen36 in a planktonic suspension. (**b**) Zoom in of bacterial clusters stained by the EL probe. Scalebars are 10 µm.

To investigate the specificity of the probe, fluorescence microscopy was applied on a planktonic bacterial suspension that was grown for 48 h with the EL probe. Figure 3 shows that the EL probe produced a fluorescence signal and was able to stain both single bacteria (Fig. 3a) and bacterial clusters (Fig. 3b). The extracellular polymeric substances of the bacteria were also stained by the EL probe (Fig. 3b).

**Fig. 4:**
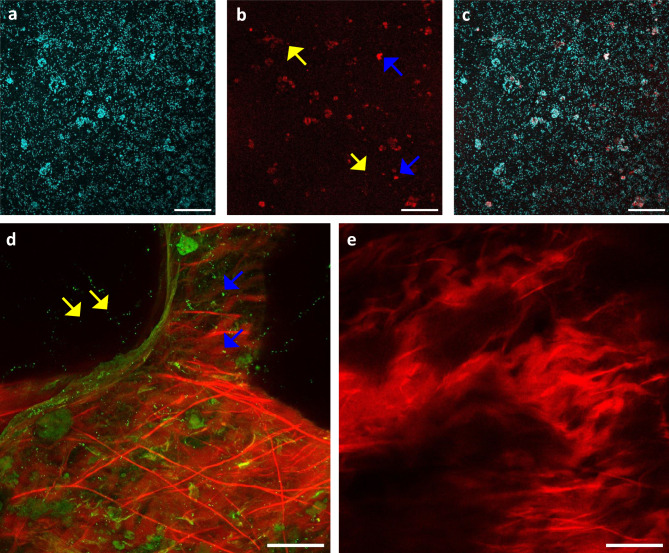
Evaluation of the EL probe specificity with confocal microscopy. (**a**–**c**) CLSM images of a 24 h S. aureus Xen36 biofilm grown in the presence of the EL probe stained with DAPI (cyan). (**a**) CLSM image of the DAPI-blue channel. (**b**) Same as panel a, but now with the EL probe red channel. Blue arrows indicate bacterial clusters and yellow arrows indicate single bacteria stained by EL probe. (**c**) The merged CLSM images of panel a and b. (**d**) CLSM image of S. aureus Xen36 stained with SYTO9 (green) grown in the presence of the EL probe (red) on a collagen-based membrane. Blue arrows indicate small bacterial clusters and yellow arrows indicate single bacteria stained by SYTO9. (**e**) CLSM image of the non-contaminated collagen-based membrane stained with the EL probe. Scalebars are 50 µm.

*S. aureus* Xen36 biofilms grown in the presence of the EL probe showed single bacteria and bacteria in clusters (Fig. 4a–c), but the probe signal appeared less bright than the fluorescence from the DAPI staining. To further assess the specificity of the probe, *S. aureus* Xen36 contaminated collagen-based membranes were assessed by confocal microscopy. Figure 4d shows that the EL probe stained collagen fibers (depicted in red), whereas for the majority of bacteria stained by SYTO9, fluorescence from the EL probe was not visible. Figure 4e shows that non-contaminated membranes were similarly stained by the EL probe.

### In vivo* EL probe sensitivity and specificity*

**Fig. 5:**
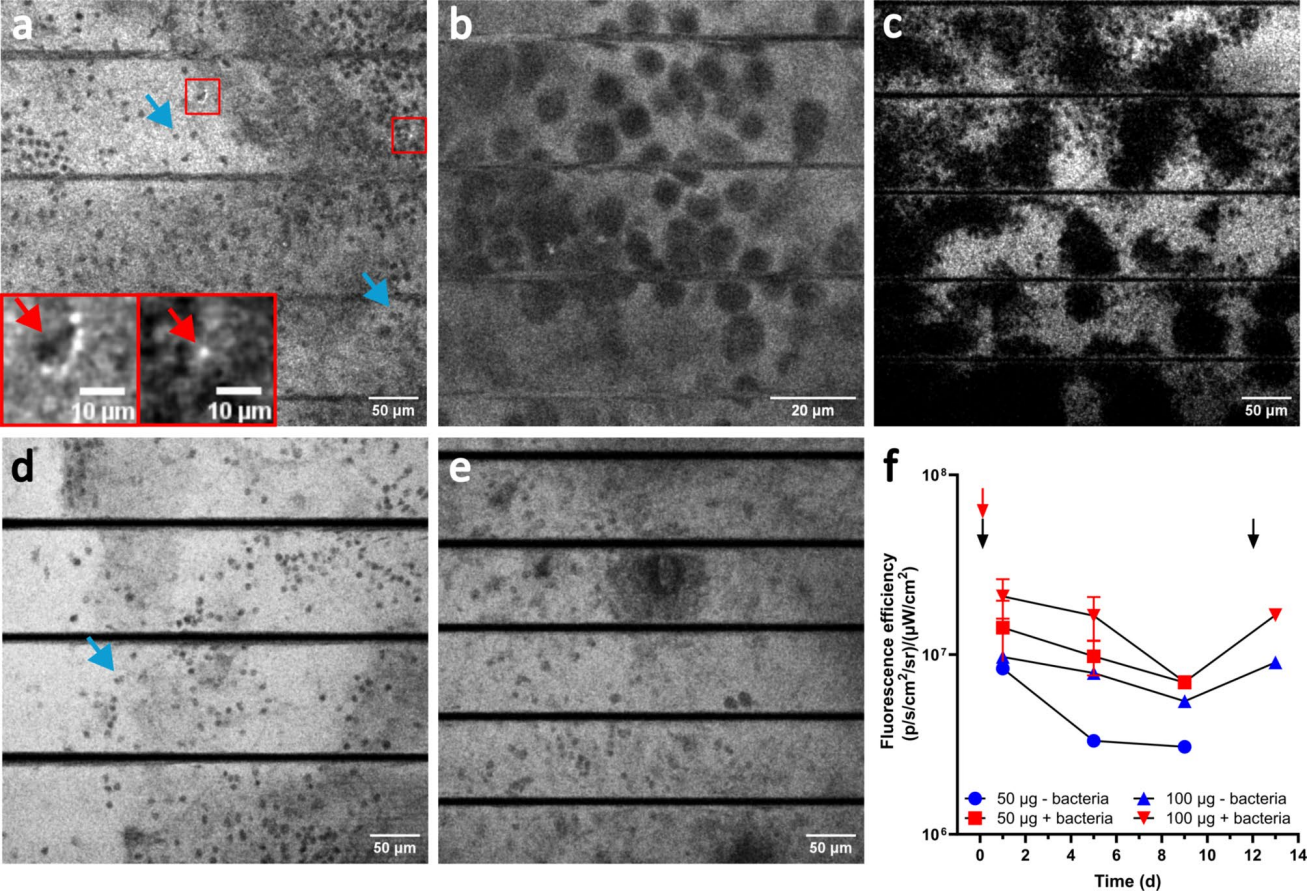
Two-photon microscopy images 20–25 µm underneath the window in the mouse shows nonspecific binding of the EL probe. (**a**) 2 days after implantation of the window and 1 day after injecting the EL probe (50 µg) and bacteria. Blue arrows indicate tissue cells (dark spots). Red arrows indicate bacteria (bright white spots). (**b**) Same condition as panel a but with higher magnification. (**c**) 6 days after implantation and 5 days after EL probe injection (100 µg) and bacteria. Panel (**d**) and (**e**) show two-photon microscopy images on day 1 with 100 µg of EL probe either with (d) or without (e) bacteria. Image gain and brightness are identical in both images. (**f**) In vivo macroscopic imaging of the mice underneath the window for either 50 or 100 µg of probe as a function of time, with or without bacteria injected. Red arrow indicates injection of bacteria on day 1, black arrows indicate injection of the probe on day 1 and day 13. n = 1 for the two concentrations without bacteria, n = 2 for 50 µg with bacteria and n = 3 for 100 µg with bacteria. Horizontal black lines in the images depict breathing artefacts, where tissue moves out of focus during breathing of the mouse.

To assess the sensitivity and specificity of the EL probe in vivo, a mouse model was applied with an imaging window serving as a foreign body towards the subcutaneous mouse tissue. Bacteria were injected underneath the window and two different doses of the EL probe were administered through the tail vein of the mouse. Using two-photon imaging 24 h after administering the probe, the extracellular matrix (ECM) surrounding the tissue cells was stained, as observed by the white or light-grey background (Fig. 5a,b). Remarkably, the tissue cells were negatively stained (blue arrows), while only a few single bacteria could be observed as bright white dots on the outside of the cells as indicated by the red arrows (see Fig. 5a, inserts). On day 6 (Fig. 5c), large unstained clusters of tissue cells were observed in black together with a more uneven pattern of ECM staining (white). Figure 5d,e show two-photon images of mice with the high dose of the probe (50 µg) in the presence (Fig. 5d) and absence (Fig. 5e) of *S. aureus*, with identical microscope settings. This illustrates that also in the absence of an infection the surrounding of tissue cells was stained, indicating that the EL probe binds to other targets different from bacteria. Macroscopic imaging showed that increasing the dose of the probe from 50 to 100 µg per animal resulted in an increase in fluorescence signal (Fig. 5f) in the presence and in the absence of bacteria. Over the course of several days the fluorescent signal decreased, while an injection with a second dose on day 13 increased the signal again. Bioluminescence of bacteria did not differ between the two concentrations of the probe in infected mice and the relationship between fluorescence and bioluminescence did not differ between infected and non-infected mice (Supplementary material, Fig. S4). In non-infected mice, administration of a high dose of the EL probe (100 µg) showed an increase in fluorescent signal using macroscopic imaging compared to administration of a lower dose of the EL probe (50 µg). These findings are consistent with the unspecific binding of the probe observed in Fig. 5a–e.

## Discussion

This study focused on the analysis of the sensitivity and specificity of the EL optotracer both in vitro and in vivo for *S. aureus* detection and infection diagnosis. Regarding the sensitivity of the probe, the fluorescence signal increased with the number of CFUs, while the EL probe adsorption to the bacterial cell wall did not inhibit cellular metabolism and growth.

These findings align with previous studies that employed optotracers with similar functionality to monitor real-time the formation of biofilms by *Salmonella enterica* serovars *Enteritidis* and *Typhimurium*^25^, and fungal biofilms of *Candida albicans*^26^. Despite the ability to perform biofilm detection, these studies, however, also showed limitations of optotracer sensitivity and specificity. Weak fluorescence signals were easily covered by stronger fluorescence generated by other probes used simultaneously with the optotracer^26^. Additionally, the risk of an unexpected binding of optotracers with unknown molecules present in the sample limited the specificity^25^. In our study we additionally found that with low amounts of bacteria (< 10^8^ CFU/ml) the fluorescence of the unbound tracer is too strong, and the enhanced fluorescence of bacterial bound tracers is insufficient to overrule the tracer background fluorescence. We further suggest that at high bacterial concentrations, depletion of non-adsorbed tracers in suspension occurs as a result of the increasing number of adsorbed tracer molecules on the expanding surface area of the cellular substrate. This changes the balance between non-adsorbed and adsorbed tracers and will result in a lower number of adsorbed tracers per bacterium. This balance is acquired fast, since we found a stable fluorescence with time. Also, tracer transport by diffusion is not a limiting factor in bacterial detection based on the immediately acquired fluorescent signal. Moreover, and in accordance with the assumption that adsorption is reversible, and taking into account competitive binding (blocking), we found that tracer adsorption can be described by a simple Langmuir type of adsorption and blocking^27^. This, however, would not rule out that increased scattering and absorption (the inner filter effect) of both excitation and emission light in concentrated suspensions of bacteria may have effect on the non-linearity of the fluorescence. Since exposure time to fluorescence excitation light is similar in all assays, we do not expect quenching to influence linearity.

These in vitro results cannot easily be translated to other strains or bacterial species. As was found earlier, EL probes are selective for Gram-positive bacteria, based on the activation of the probe when exposed to peptidoglycan and/or lipoteichoic acid, both constituents of gram-positive bacterial cell membrane^1^. This was re-established with two other Gram-positive strains (*S. aureus* 12600 and *S. epidermidi*s ATCC 35984) and in comparison, a Gram-negative strain *E. coli.* ATCC 25922 (Supplementary material, Fig S3), clearly showing this selectivity of the probe. Although the probe appeared sensitive to other species (*S. epidermidis*) and *S. aureus* strains, the adsorption characteristics (as e.g. *Γ*_*max*_*)* may differ, as the available adsorption sites are strongly dependent on the architecture of the bacterial cell envelope^1^.

Although we presented data for both planktonic and a biofilm mode of growth, the setup of the assay may have an effect on the results as well. Currently, several methods exist for culturing bacterial biofilms, which can differ in environmental conditions like temperature, flow rate, nutrient exchange, shear stress, having an effect on the permeability of the biofilm^28,29^. In this study the bacterial biofilms were grown under static conditions; it is reasonable to expect that the conclusions drawn on the sensitivity and specificity of the probe in vitro may differ under different environmental conditions.

By imaging bacteria in suspension by fluorescence microscopy, we found that *S. aureus* bacteria present as clusters or aggregates in a cloud of stained extracellular material. Although EPS is mainly produced in a biofilm and not by planktonic bacteria^30^, bacterial aggregates may also excrete EPS^31^ and various virulence factors^32^. These results are in agreement with previous studies which have highlighted that the EL probe is able to stain the amyloid protein curli from *Salmonella* bacteria, characterized by repetitive beta-sheet motifs^33^. Although *S. aureus* does not produce these amyloid proteins, excreted polymeric molecules in aggregates of *S. aureus* contain a wide combination of proteins and biopolymers such as proteases and fibronectin-binding proteins that may contain beta-sheets as secondary structures and may form a substrate for the optotracer activation^34,35^.

Regarding specificity, the in vivo results closely mirrored the in vitro findings with the collagen-based membrane. In vivo, we found a remarkable positive staining of the ECM with non-stained “negative cells”, clearly witnessing that the EL probe does not pass the cell membrane of the cells infiltrated in the vicinity of the implanted window. Macroscopic imaging of mice with and without bacterial infection showed only marginal differences in fluorescence between the two conditions, which indicates that EPS proteins are not the sole contributors to the non-specific staining by the EL probe; extracellular matrix (ECM) proteins are also stained. A second administration of the probe increased the fluorescence in both conditions, showing that the signal is indeed generated by the EL probe. Still, and in line with the marginal differences in macroscopic imaging findings, we observed a limited number of positively stained individual bacteria by two-photon microscopy, 24 h after probe injection. The low number of positively stained bacteria probably is a result of the probe depletion due to the extensive staining of the ECM, similar to our in vitro results observed with high amounts of bacterial cells (> 10^9^). These results reflect the conclusion that the EL probe shows insufficient specificity, and as a result also loses sensitivity, in vivo. It should be noted that the number of mice in this study was limited, making it difficult to draw robust conclusions about the sensitivity of the probe in vivo. However, this reflects the proof-of-concept character of this study for the use of the EL probe in vivo, aiming to investigate the specificity of the EL probe.

It is unclear which ECM molecules are stained in particular, except for bacterial EPS and collagen fibers that are abundantly present in ECM. As optotracers most likely bind to the β (1–4) linkage of disaccharides in the peptidoglycan layer of the cell wall of Gram-positive bacteria^17^, it is likely that they will also bind to other disaccharides with a similar linkage. In mammalian cells, many glycosaminoglycans (GAGs) contain these β (1–4) linkages^36^ such as hyaluronan, a major component of the ECM^36^. Other GAGs, such as chondroitin sulfate, play a role in inflammation through interaction with chemo- and cytokines^36^. It is therefore expected that the EL probe will bind these GAGs that are either a component of the ECM or play a role in the inflammation induced by the imaging window. Another possibility is the binding of the EL probe to amyloid-like aggregates that resemble bacterial amyloids present in Gram-positive bacteria^37^. Apolipoprotein A (APOE) plays a role during inflammation and in the host response to *S. aureus*, and has been shown to form amyloid-like aggregates which could be detected by a different fluorescent optotracer^38–40^. Taken together, the obtained results suggest that the EL probe binds to repetitive component motifs that are both present in bacterial EPS as well as in the ECM of the inflamed or infected tissue.

## Conclusion

We have evaluated the performance of the optotracer EL for bacterial detection of the bioluminescent strain *S. aureus* Xen36, both in vitro and in vivo. Our main conclusion establishes a solid foundation for the quantitative use of the EL probe in planktonic suspensions and biofilms in vitro. However, when applying the probe for bacterial quantification, particularly for *S. aureus*, it is important to consider the CFU-fluorescence relationship. Furthermore*, *in vivo results indicate that the probe binds to repetitive component motifs that are abundant in bacterial extracellular polymeric substances (EPS) and tissue extracellular matrix (ECM). Therefore, caution is advised when using this probe in vivo, as a strong background signal may obscure bacterial detection.

## Materials and methods

### Bacterial growth and harvesting

Bioluminescent *S. aureus* Xen36 (PerkinElmer Inc., Waltham, USA) was cultured from cryopreservative vials on Tryptone Soya Agar (TSA) (Oxoid Ltd., Basingstoke, UK, Fisher Scientific Int., Hampton, USA) plates with 200 µg/ml kanamycin (Sigma-Aldrich, St. Louis, USA) and incubated at 37 °C overnight. *S. aureus* ATCC 12600, *S. epidermidis* ATCC 35984 and *E. coli* ATCC 25922 were cultured on blood agar (Xebios Diagnostics Group, Düsseldorf, Germany) and incubated at 37 °C overnight. One colony was inoculated in 10 ml of Tryptone Soya Broth (TSB) (Oxoid Ltd.), for *S. aureus* Xen36 supplemented with 200 μg/ml kanamycin, at 37 °C while shaking at 150 rpm for 24 h. For the main culture, 10 ml of the preculture was put in 200 ml of fresh TSB and grown for 16 h at 37 °C, while shaking at 150 rpm. The main culture was centrifuged three times at 5000 g at 10 °C, for 5 min and resuspended in phosphate buffered saline (PBS, 10 mM potassium phosphate, 150 mM NaCl, pH 7.0). The bacterial concentration was determined with the Bürker-Türk counting chamber and diluted to the concentration necessary for the different assays.

### Sensitivity and adsorption of the EbbaBiolight probe

The EbbaBiolight 680 (EL) probe was obtained from EbbaBiolight (Ebba Biotech AB, Stockholm, Sweden) at a concentration of 1 mg/ml in ultrapure water. The EL probe was diluted to 1 μg/ml in PBS + 2% TSB. Different initial inoculums of 3 × 10^10^, 10^10^, 10^9^, 10^8^, 10^7^ and 10^6^ bacteria/ml were resuspended in PBS + 2% TSB with 1 μg/ml of EL probe, a medium with sufficient nutrients for bacteria to survive without allowing reproduction. Then, 100 µl of each suspension was added into a well of a 96 black-well plate and fluorescence was measured with the plate reader with the following settings: excitation wavelength 540 nm, emission wavelength 680 nm, gain 100. Fluorescence of the bacterial suspension with the probe was measured at each hour during 8 h and after 8 h CFUs were determined by serial dilutions and agar plating. As a negative control the same bacterial suspension without the probe was used.

For inter-species and -strain comparisons, bacterial stock solutions were first diluted to 2 × 10^10^ CFU/mL in PBS to create equal stock solutions and subsequently diluted to 10^10^ bacteria/mL in PBS + 2% TSB with 1 μg/ml of the EL probe. Of this solution, 100 μl was added to a 96 black-well plate and fluorescence was measured immediately after using the same parameters as described above.

The total fluorescence, φ_total_, of the bacterial suspensions with the EL-probe was fitted against:

1 $$\varphi_{total} = \, \varphi_{unbound} + \, \left( {N_{bact} * \, \varphi_{bound} } \right)^{n}$$

with *φ*_*unbound*_ corresponding to the fluorescence of the total amount of probe in the suspension and not bound to the bacterial cell surface and *φ*_*bound*_ corresponding to the enhanced fluorescence from the probe that did bind to a single bacterium. *N*_*bact*_ is the number of bacteria in suspension and the factor n indicates the power that in case it is unequal to 1, may compensate for non-linearities.

The EL probe concentrations of 0.01; 0.1; 1; 100 μg/ml were diluted in PBS + 2% TSB and bacterial suspension in PBS + 2% TSB was added with a final concentration of 10^10^ bacteria/ml. Then, 100 µl of each of these bacterial suspensions with probe was transferred to a 96 black well plate. As a negative control the bacterial suspension without the probe was used. The probe concentration data were used to construct a Langmuir adsorption isotherm that is assumed typical for the adsorption process of molecules to a substrate. For low concentrations of the probe in the suspension or solution this adsorption isotherm was previously described^27^ as the following equation:

2 $$\frac{\theta }{1-\theta }=KX$$

with *X* as the concentration of the unbound probe in suspension, *K* as an equilibrium constant and *θ* is defined as:

3 $$\theta (X)=\frac{\Gamma (X)}{{\Gamma }_{max}}$$

with *Γ(X)* and ${\Gamma }_{max}$ the actual adsorbed probe concentration and the highest probe concentration, respectively, on the bacterial cell surface. From Eq. 2 it can be inferred that the adsorbed amount of the probe is not linearly correlated to the concentration of unbound probe in the suspension. Assuming, however, that the fluorescence is linearly correlating with the concentration of the adsorbed probes and supersedes the fluorescence of the unbound probe (as is true for high bacterial concentrations), we took the fluorescence as a value for *Γ*. The value of ${\Gamma }_{max}$ is not known beforehand, therefore we fitted our concentration assay data to Eq. 1 to obtain a linear function.

### Bacterial growth with the EL probe in the planktonic and biofilm mode

*S. aureus* Xen36 was suspended in TSB at 10^6^ bacteria/ml or 10^8^ bacteria/ml with 1 μg/ml of the EL probe. Then, 100 µl of each bacterial culture was transferred to a 96 black well plate (Greiner Bio-One, Kremsmünster, Austria) and grown for 48 h at 37 °C. The bacterial suspensions without the EL probe were used as a negative control. Bioluminescence and fluorescence measurements were executed and CFU counting was performed using agar plating after serial dilution. In addition, fluorescence microscopy was performed after 48 h.

For biofilm formation, an initial inoculum of 100 µl of the *S. aureus* Xen36 (10^8^ bacteria/ml) suspension in PBS + 2% TSB was placed in a 96 black well plate. After 2 h of bacterial adhesion at 37 °C, the solution was carefully removed only leaving the bacteria adhered to the surface of the well. 100 µl of fresh TSB with or without 1 μg/ml of the EL probe was added to the wells and the well plate was incubated at 37 °C for 48 h. The fluorescence was measured on defined timepoints where the addition of the probe was considered as the 0 h timepoint.

For bacteria, both in the planktonic and biofilm mode of growth, CFU counting, bioluminescence and fluorescence measurements were performed at 0, 8, 24 and 48 h as described below. Three biological replicates were carried out for the entire study. For the CFU counting, the bacterial samples were serially diluted using a tenfold dilution in PBS and three 10 µl drops of each dilution were placed on TSA plates. For the biofilm assay, adherent bacteria were scraped from the well surface using a sterile pipet tip. To disrupt bacterial clusters, the solution was collected in an Eppendorf tube and sonicated for 5 min using a sonication bath. Plates were incubated for 20 h under aerobic conditions at 37 °C after which colonies were counted.

### Bioluminescence and fluorescence analysis

Bioluminescence from *S. aureus* Xen36 in wells in both the planktonic and biofilm suspensions were measured at 0, 8, 24 and 48 h using the in vivo imaging system (IVIS) Lumina (PerkinElmer, Waltham Massachusetts, USA). The data were analyzed using the LivingImage 4.5.5 software (PerkinElmer). A field of view of 12.5 × 12.5 cm (sufficient for capturing an image of all wells simultaneously) and 2-min excitation were used.

Fluorescence from the activated EL probe in wells in both the planktonic and biofilm cultures were measured at 0, 8, 24 and 48 h using the microplate reader.

### Microscopy imaging

Confocal imaging was performed on a 24 h biofilm which was cultured in the presence of the EL probe (concentration of 1 μg/ml) using a confocal laser scanning microscope Stellaris 5 (Leica, Wetzlar, Germany) with the LAS X software and a 40X objective in water. Prior to imaging, biofilms were stained with DAPI (D9542, Sigma-Aldrich, St. Louis, UK) according to the manufacturer’s indications. Images were subsequently analyzed using the ImageJ software.

Fluorescence microscopy was performed to image bacteria in suspension. Therefore 20 μl of the *S. aureus* Xen36 with an initial inoculum of 10^8^ bacteria/ml cultured over 48 h was placed on a microscope slide and covered with cover slip (Thermo Fisher Scientific, Waltham, USA). Microscopy was performed using a Leica fluorescence microscope DM4000 B (Leica) applying the 100X objective in oil and a gain of 50% with LAS V4.12 software. Images were analyzed using the ImageJ software.

### Collagen membranes

To test the EL probe specificity to bacteria, collagen-based membranes (Meso Wound Matrix™ Scaffold, DSM, Heerlen, Netherlands) were cut and glued to the bottom of a 6 well plate. Each membrane was immersed for 24 h in 2 ml with 1 μg/ml of EL probe in TSB. Subsequently, CLSM imaging of the membranes was performed (Fig. 4e). In a subsequent experiment, the protocol of biofilms in contact with the membrane was followed. Briefly, the membranes were cut and glued to the bottom of the 6 well plate and 2 ml bacteria (10^8^ bacteria/ml) in PBS + 2% TSB were put on top of the membrane and incubated for 2 h to let the bacteria adhere. Subsequently, the solution was removed and the membrane was immersed in 2 ml with 1 μg/ml of EL probe in TSB and incubated for 24 h. Then, the bacterial DNA was stained with SYTO9 (Thermo Fisher Scientific, Waltham, USA) according to the manufacturer’s indications and confocal imaging was performed.

### Ethical approval

Animal experiments were approved by the Central Committee for animal experiments of the Dutch government (Approval number AVD20500202317088). All experiments were performed in accordance with the relevant guidelines and regulations and in compliance with the ARRIVE Guidelines 2.0.

### Animals

In total, 9 C57BL/6J female mice (Charles River, France) of approximately 20 g (8–12 weeks) were used. Female mice were chosen due to the lower frequency of fighting compared to male mice, which reduces the loss of window due to fighting. Upon arrival, animals were acclimated for 1 week in individually ventilated cages. No animals were excluded before the start of the experiment, based on weight and physical appearance. Throughout the experiments, mice were kept on an alfalfa-free diet to reduce auto-fluorescence. Animals were examined every 2 days, noting aberrations in behavior and signs of local infection. During procedures, anesthesia was induced using 5% isoflurane in oxygen and maintained at 2% isoflurane in oxygen. Four different conditions were tested: 2 different concentrations of the EL probe (50 and 100 µg), both with and without a bacterial infection. Doses were chosen based on company recommendations, where a dose of 100 µg has been tested for toxicity. Moreover, in vivo testing in two mice with a dose of 10 µg did not yield any signal (data not shown). Mice were randomly assigned to each group. Each condition included a single mouse that remained in the experiment until day 14. Three infected mice were followed up until day 6, 1 with 50 µg of the EL probe and 2 with 100 µg of the EL probe. After the procedures animals were killed by cervical dislocation as prescribed in Annex IV of Directive 2010/63/EU.

### Window implantation in mice

Windows were assembled using 13 mm coverslips and titanium rings. Coverslips were cleaned with 2% Extran® and 70% ethanol and mounted in titanium rings using cyanoacrylate glue. The windows were subsequently sterilized using 4 kGy gamma irradiation. Briefly, isoflurane anesthetized mice were shaved on their right flank, placed on a heating pad and eyes were lubricated with eye ointment. The skin was disinfected with 0.5% chlorhexidine. A 17 mm lateral incision was made in the dorsolateral portion of the skin, starting immediately below the lower rib, without cutting through the underlying fascia. The skin was then separated from the underlying fascia by blunt dissection. The tissue was kept wet using 0.9% NaCl. A purse string suture was made around the edge of the incision using a 5–0 perma-hand silk black braided suture (N266H, Ethicon, Raritan, USA). The window was placed in the incision and the suture drawn tight and fixed. Subcutaneous 0.05 mg/kg buprenorphine was administered for pain relief and mice were allowed to recover on a heating pad. *S. aureus* Xen36 was cultured as described above. On day 1 after window implantation (experiment day 0), 20 µL bacterial suspension (5 × 10^8^ CFU/ml) in PBS was injected directly underneath the window using ½U Micro-Fine insulin needles (0.3 ml, Becton Dickinson, Franklin Lakes, USA) under anesthesia. Location of the inoculation was confirmed visually, observing the needle position through the window. One day before two-photon microscopy (day 0, 4, 8 and 12), animals were injected in the tail vein with 100 µl of EL probe solution in PBS with different concentrations (500 or 1000 µg/ml) which is approximately 1 or 2 mg/kg bodyweight.

### In vivo* macroscopic imaging*

The IVIS Lumina was used to assess infection persistence by measuring bioluminescence and distribution of EL probe by measuring fluorescence on days 2, 6, 10 and 14. For imaging, 2 mice were placed simultaneously in a prewarmed IVIS chamber under 2% isoflurane anesthesia. Mice were positioned with their nose in the anesthesia tube and the window exposed to the camera. After obtaining grey-scale photographs, bioluminescence images were taken using a 12.5 × 12.5 cm FOV, with 5 min exposure time, binning 4 and 1/f aperture. Fluorescence images were taken using a 12.5 × 12.5 cm FOV, binning 2, 2/f aperture, 1 s exposure time, excitation at 535 nm and emission filter at 810–875 nm, as optimized for the EL probe with this machine. Regions of interest were manually drawn on each mouse at the location of the glass section of the window and photon fluxes per radiance were obtained using Living Image software 4.8.0 (PerkinElmer, Waltham, USA).

### Intravital two-photon confocal microscopy

The applicability of the EL probe in intravital microscopy in the mouse model was assessed using two-photon confocal microscopy. After induction of anesthesia using 5% isoflurane in oxygen, mice were placed in a holder where the window was clamped to minimize movement artefacts. Mice were then transferred in the holder to the pre-warmed, closed microscope stage where maintenance anesthesia was used for a maximum duration of 45 min of imaging. Two-photon microscopy was performed using a Zeiss LSM 7MP microscope (Zeiss, Oberkochen, Germany) coupled to a Chameleon Vision compact OPO two-photon laser and a W plan-Apochromat 20x/1.0 DIC (WD = 1.8 mm) objective, (421452–9900, Zeiss). EL probe was imaged using an excitation wavelength of 800 nm and an emission filter of 650–705 nm. Z-stacks of 5 µm images were generated up to 200 µm underneath the glass.

### Statistical analysis

Statistical analysis was performed using GraphPad Prism 8.0.1 (GraphPad, San Diego, United States). Statistical differences were calculated using multiple t-test and P < 0.05 was considered significant. For the in vitro part, three biological replicates were carried out for the entire study.

## Supplementary Information

Supplementary Information 1.

Supplementary Information 2.

Supplementary Information 3.

Supplementary Information 4.

Supplementary Information 5.

## Data Availability

Data is available on request from the corresponding author.
