# Supplementary figures and images for: The applicability of fluorescent optotracers for in vitro and in vivo Staphylococcus aureus detection and quantification
Source: Sci Rep. 2025 Oct 3;15:34503. doi: 10.1038/s41598-025-17029-7 (PMC12494981; doi:10.1038/s41598-025-17029-7)

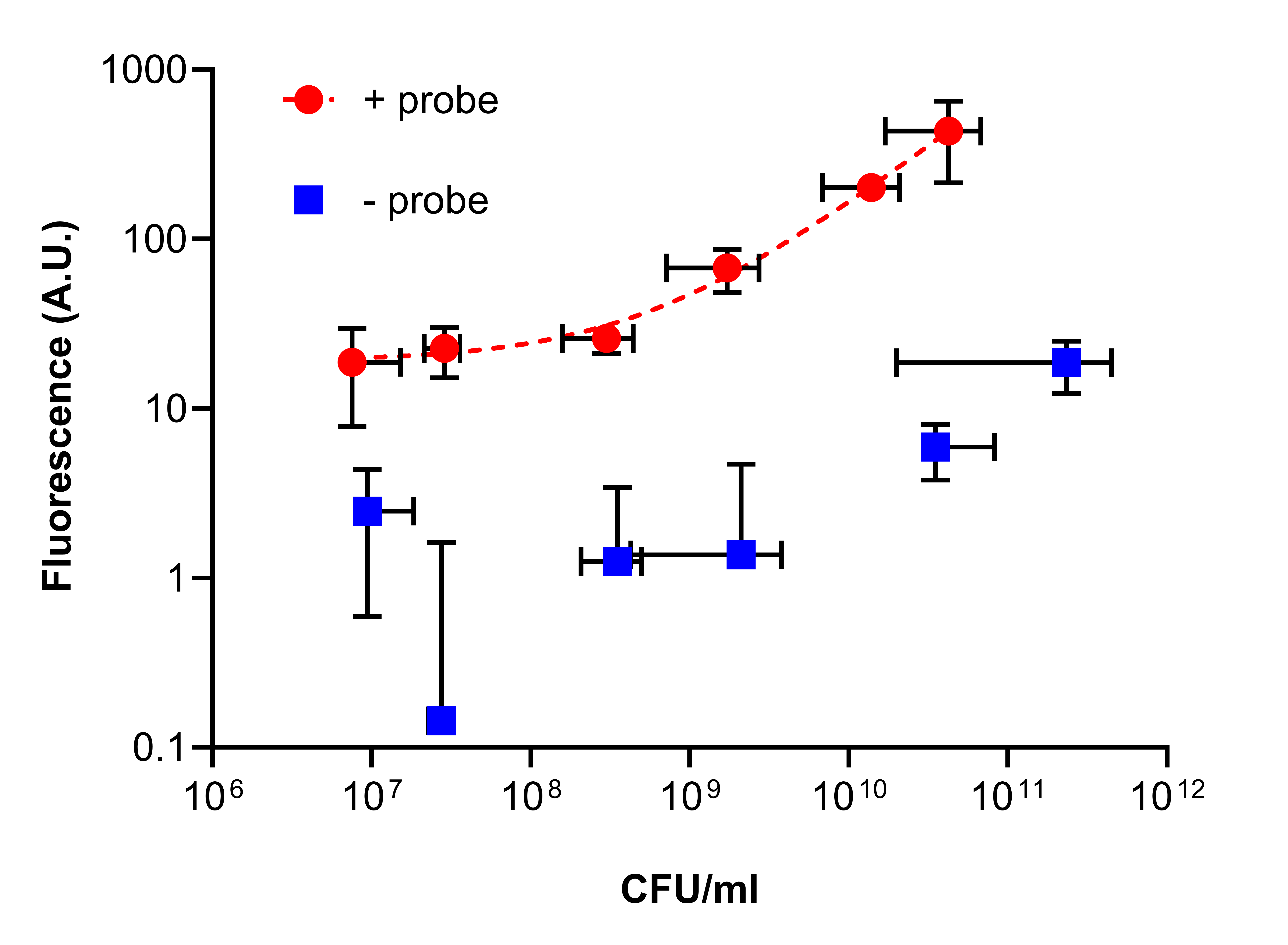

Supplement: Supplementary file 1 — Supplementary Information 1. [file 41598_2025_17029_MOESM1_ESM.tif]

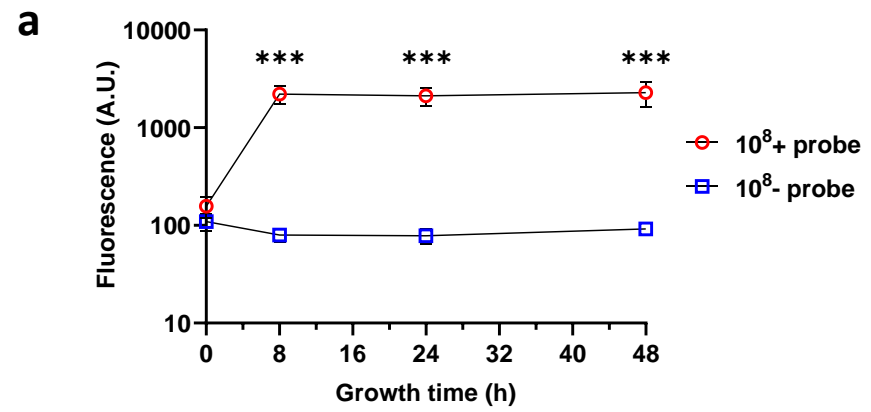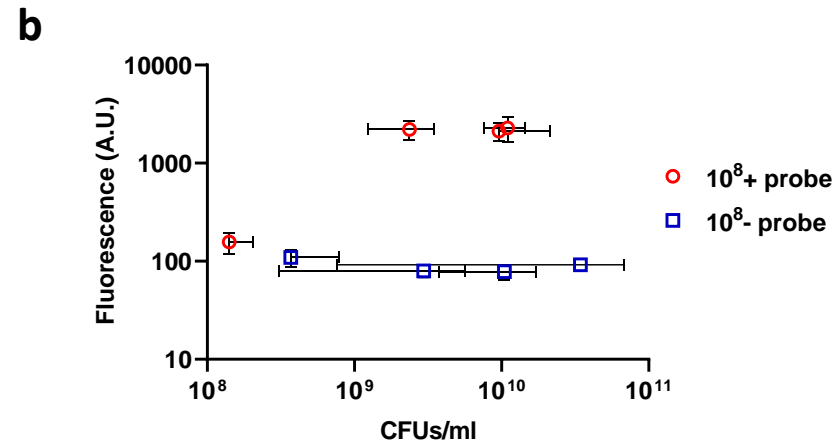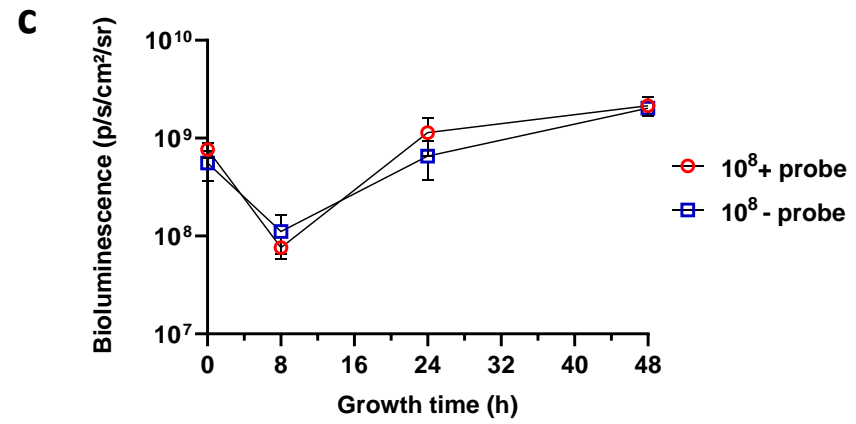

Supplement: Supplementary file 2 — Supplementary Information 2. [file 41598_2025_17029_MOESM2_ESM.pdf]

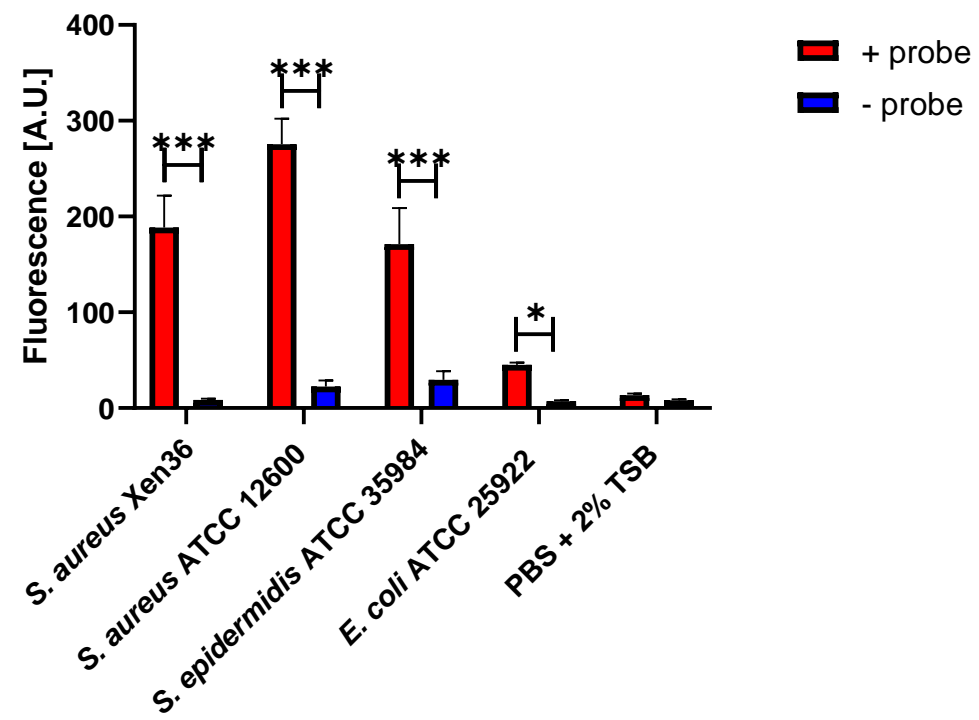

Supplement: Supplementary file 3 — Supplementary Information 3. [file 41598_2025_17029_MOESM3_ESM.pdf]
