# Supplementary material for: The applicability of fluorescent optotracers for in vitro and in vivo Staphylococcus aureus detection and quantification
Source: Sci Rep. 2025 Oct 3;15:34503. doi: 10.1038/s41598-025-17029-7 (PMC12494981; doi:10.1038/s41598-025-17029-7)
Supplement: Supplementary file 5 — Supplementary Information 5. [file 41598_2025_17029_MOESM5_ESM.docx]

**Supplementary material**

Belonging to:

**The applicability of fluorescent optotracers for *in vitro* and *in vivo* *Staphylococcus aureus* detection and quantification**

Liliana Agresti^1^, Elles C. Boonstra^2^, Paul C. Jutte^2^, Henny C. van der Mei^1^, Jelmer Sjollema^1^

*
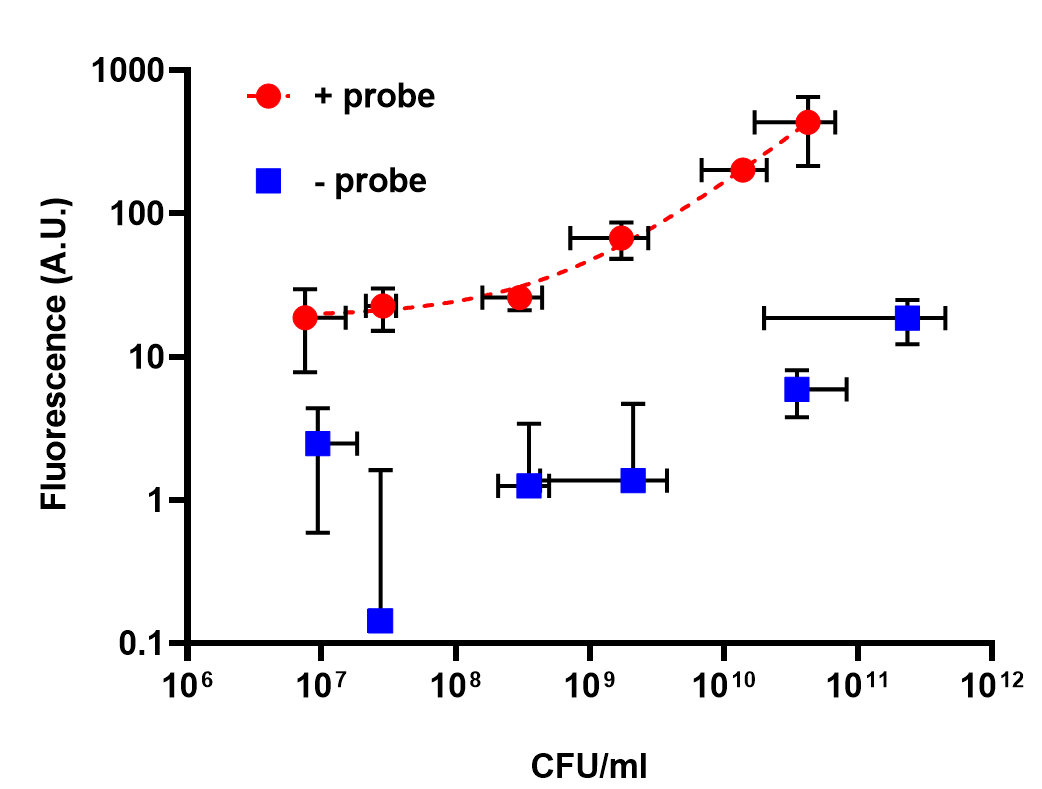
*

*Figure S1: Fluorescence of planktonic bacteria in PBS with 2% TSB with (red) or without (blue) the EL probe (1 μg/ml) as a function of the concentration of bacteria after 8 h of incubation. The dotted red line is a regression line, fitted to φ_total_= φ_unbound_ + (N * φ_bound_ )^n^ with φ_total_ the fluorescence from the suspension with the EL probe, φ_unbound_ corresponding to the fluorescence of the total amount of probes that are in the suspension and not bound to the bacterial cell surface and φ_bound_ corresponding to the enhanced fluorescence from probes that adsorbed to a single bacterium. N is the number of CFU/ml and n=0.7 is the factor of the power that in case it is unequal to 1 may compensate for non-linearities. R^2^=0.75. This graph represents similar data as presented in Fig. 1b but on a log-log scale.*

*
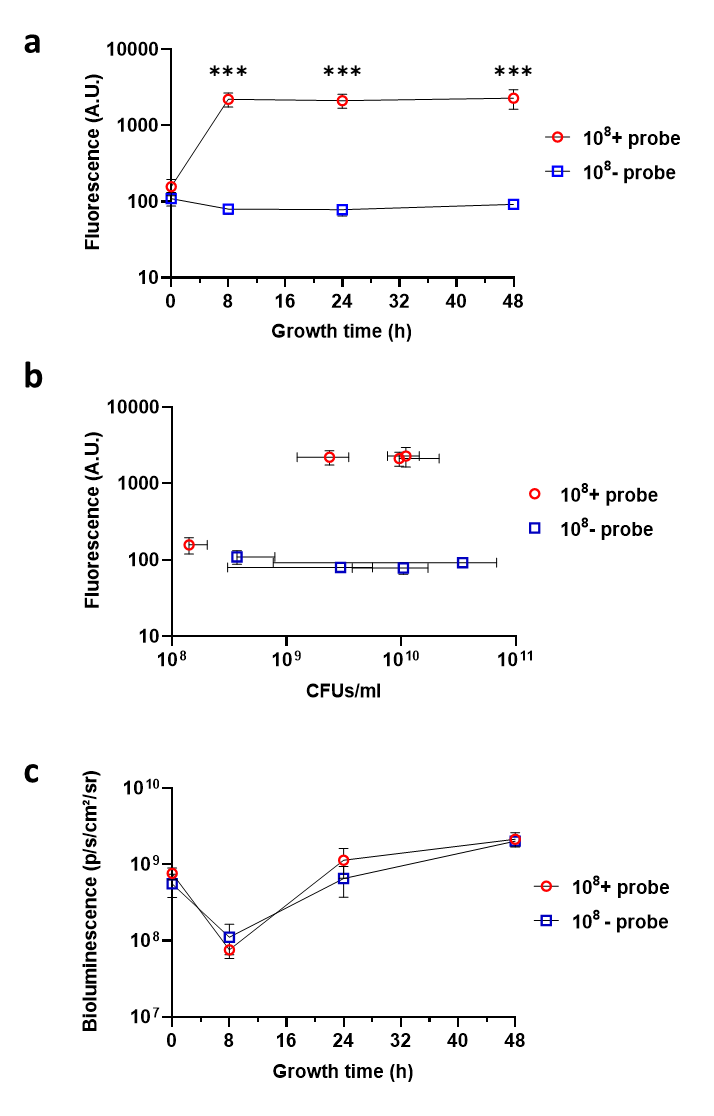
*

*Figure S2: EL Probe sensitivity: a) Fluorescence of the EL probe (1 μg/ml) as a function of growth for planktonic bacteria with an initial inoculum of 10^8^ CFU/ml. b) Same as panel a, now for CFU/ml from the samples taken at the same time points as panel a****.*** *c) Same as panel a, now for the bioluminescence of* the S. aureus *Xen36 as a function of growth. Statistical differences were calculated using multiple t-tests and the differences with the control without the probe were considered statistically signiﬁcant when P<0.05. *** when P<0.001.*

*Figure S3: EL probe sensitivity with different bacterial strains. Fluorescence of the EL probe (1 μg/ml) with different bacterial strains diluted to 10^10^ bacteria/mL in PBS + 2% TSB. Fluorescence measurements were taken after the injection of the EL probe with gain 100. Statistical differences were calculated using multiple t-tests and the differences with the control without the probe were considered statistically signiﬁcant when P<0.05. *** when P<0.001, number of biological replicates = 3.*

**

*Figure S4: Bioluminescence as a function of fluorescence of the EL probe in vivo. Each point indicates a single measurement in a single mouse. Values were taken of the same region of interest of the area exposed by the imaging window.*
